# Glycine Insertion Makes Yellow Fluorescent Protein Sensitive to Hydrostatic Pressure

**DOI:** 10.1371/journal.pone.0073212

**Published:** 2013-08-27

**Authors:** Tomonobu M. Watanabe, Katsumi Imada, Keiko Yoshizawa, Masayoshi Nishiyama, Chiaki Kato, Fumiyoshi Abe, Takamitsu J. Morikawa, Miki Kinoshita, Hideaki Fujita, Toshio Yanagida

**Affiliations:** 1 RIKEN Quantitative Biology Center (QBiC), Suita, Osaka, Japan; 2 PRESTO, Japan Science and Technology Agency, Kawaguchi, Saitama, Japan; 3 WPI, Immunology Frontier Research Center, Osaka University, Suita, Osaka, Japan; 4 Graduate School of Frontier Bioscience, Osaka University, Suita, Osaka, Japan; 5 Department of Macromolecular Science, Graduate School of Science, Osaka University, Toyonaka, Osaka, Japan; 6 The HAKUBI Center for Advanced Research/Institute for Integrated Cell-Material Sciences, Kyoto University, Kyoto 606-8501, Japan; 7 Japan Agency for Marine Earth Science and Technology, Yokosuka, Kanagawa, Japan; 8 Department of Chemistry and Biological Science, College of Science and Engineering, Aoyama Gakuin University, Sagamihara, Kanagawa, Japan; CNR, Italy

## Abstract

Fluorescent protein-based indicators for intracellular environment conditions such as pH and ion concentrations are commonly used to study the status and dynamics of living cells. Despite being an important factor in many biological processes, the development of an indicator for the physicochemical state of water, such as pressure, viscosity and temperature, however, has been neglected. We here found a novel mutation that dramatically enhances the pressure dependency of the yellow fluorescent protein (YFP) by inserting several glycines into it. The crystal structure of the mutant showed that the tyrosine near the chromophore flipped toward the outside of the β-can structure, resulting in the entry of a few water molecules near the chromophore. In response to changes in hydrostatic pressure, a spectrum shift and an intensity change of the fluorescence were observed. By measuring the fluorescence of the YFP mutant, we succeeded in measuring the intracellular pressure change in living cell. This study shows a new strategy of design to engineer fluorescent protein indicators to sense hydrostatic pressure.

## Introduction

Fluorescent protein, which is perhaps the most popular fluorescent probe in life science due to its simple and easy labeling, is a spontaneous fluorescent protein isolated from Pacific jellyfish ( *Aequoria* *victoria* ) [1–3], and other fluorescent proteins exhibiting various emission spectra have been engineered by means of direct mutagenesis, and/or isolation from different coelenterates [4,5]. In conjunction with different molecular biology techniques such as direct mutagenesis, circular permutation and Förster resonance energy transfer, various fluorescent proteins have been developed to study the effects of intracellular properties including pH, Ca^2+^-concentration, and tensile force within a protein, on cellular behavior [6–9]. Despite their importance in numerous cellular processes, fluorescent proteins have not proven usable for measuring the physicochemical state of water, which affects protein functions, i.e., the enzymatic activity and structural stability of a protein strongly depend on the temperature and/or pressure of the solution [10–12]. Although the fluorescence intensity of fluorescent protein actually depends on the temperature and hydrostatic pressure, the intensity changes are too small to measure [13,14]. Most recently, temperature in a cell was successfully measured on a microscope using the temperature dependency of fluorescence anisotropy of green fluorescent protein (GFP) [15]. We here aimed to visualize the other state of water, hydrostatic pressure, in living cells using fluorescence reporter.

Fluorescent proteins share a common structure composed of an eleven-stranded β-barrel containing a chromophore that is spontaneously formed through cyclization of a tri-peptide sequence following the central α-helix [3,5]. Water molecules around the fluorescent protein chromophore are known to affect its characteristics including absorption and emission fluorescent spectra and intensity [16–18]. The β-barrel structure prevents the chromophore from interacting with the solvent molecules [3,5]. It is expected that structural disruption of the β-barrel structure would increase the accessibility of water molecules to the chromophore and enhances the pressure dependency of the fluorescence including the spectrum and intensity. However, the structural disruption should be slight because the solvent interaction to the chromophore causes the photo quenching. The key strategy is to increase the solvent interaction while maintaining observable degree of fluorescent intensity. In other words, we designed a fluorescent protein that has a space for a few water molecules near the chromophore.

**Figure 1:**
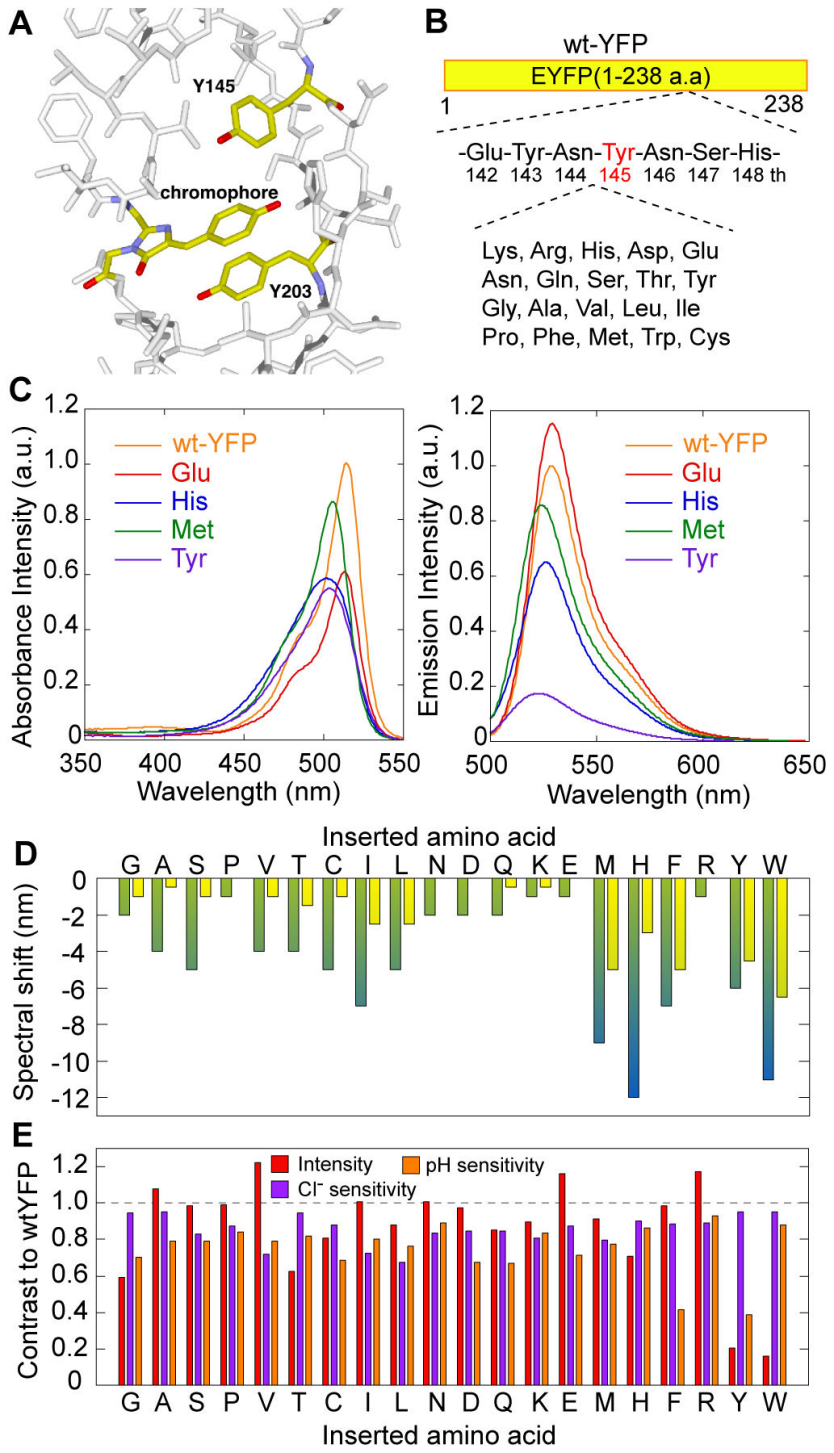
One amino acid insertion into YFP. (A) Structure around the chromophore of YFP (yellow; PDB ID: 1yfp). Red, oxygen. Blue, nitrogen. (B) Schematic drawing of the one amino acid insertion method. (C) Absorbance (left) and emission (right) spectra of the YFP mutants. Orange, wild-type YFP; red, glutamate insertion; blue, histidine insertion; green, methionine insertion; magenta, tyrosine insertion. Emission spectra were obtained at 488 nm excitation. The intensities are normalized as to the peak intensity of wild-type YFP. Concentrations are constant among all data. (D) Spectral shifts of the peak of absorbance (green) and emission (yellow) spectra of the YFP mutants. Lower alphabets stand for the inserted amino acid. Amino acids are arranged according to the van der Waals radius. (E) Fluorescence intensity (red), chloride sensitivity (magenta), and pH sensitivity (orange) of the YFP mutants. The values are normalized as to those of wild-type YFP. Lower alphabets stand for the inserted amino acid. The chloride sensitivity is defined as the ratio of the fluorescence intensities at 0 mM and 200 mM KCl. The pH sensitivity is defined as the ratio of the fluorescence intensities at pH 8.0 and 7.0.

We here focused on Tyr145 in β-strand 7 of fluorescent protein from *Aequorea* *Victoriae* , because its side chain phenol group is located adjacent to the chromophore in the center of the β-barrel structure (Figure 1A) [3,5]. We expected that the orientation change of the position of Tyr145 allows the appropriate invasion of the water molecules into the β-barrel structure, resulting in the enhancement of the pressure dependency. In this study, we selected amino acid insertion instead of substitution that has been widely used [19]. This paper shows that an effective insertion produces a dramatic structural change near the chromophore and makes a fluorescent protein sensitive to hydrostatic pressure.

## Results

### Single residue insertion into the β-barrel structure of yellow fluorescent protein

Our first design is to introduce a small orientation change at the position of the phenol group of Tyr145. We inserted an amino acid residue just before Tyr145 in order to produce a small space for water molecule to interact with the chromophore (Figure 1B). We here used yellow fluorescent protein (YFP), which is a GFP variant that has a chromophore composed of the GFP chromophore and the phenol group of Tyr203 [5,20]. The emission spectra of YFP shifts toward red depending on the stacking distance between the chromophore and the phenol group, and can be altered by hydrostatic pressure and freezing [21,22]. If an insertion can yield a space near the chromophore as we expected, the chromophore would move slightly toward the newly created space, causing a spectral blue-shift.

All 20 amino acids were examined and their fluorescence characteristics were measured (Figure 1, C-E). The insertions caused a spectral blue-shift in the absorbance and fluorescence spectra (Figure 1, C and D). The extent of the blue-shift roughly depended on the van der Waals radius, but the amino acids that have a carboxyl, an amide or an amino group (Asp, Asn, Glu, Gln, Lys, or Arg) caused little change (Figure 1D). Sensitivity to the chloride ion was somewhat augmented in those amino acids possessing an aliphatic side chain group (Leu, Ile, or Val), as the fluorescence intensity deceased ~30% when adding 200 mM KCl, but in all other cases, the fluorescence intensity was maintained above 80% (Figure 1E, *magenta*). The pH dependency remarkably increased with the amino acids that have a phenyl ring (Phe, Tyr) (Figure 1E, *orange*). The fluorescence intensity would decrease if the appropriate insertion made additional water molecules interact with the chromophore. Fifteen of the 20 amino acids kept the fluorescence intensity above 80% compared to wild-type YFP, and some of them (Ala, Val, Glu, and Arg) improved (Figure 1E, *red*). Glycine, which has no side chain, caused a 1 nm spectral blue-shift in emission and decreased the fluorescence intensity. On the contrary, alanine, whose side chain comprises a methyl group, showed only a slight spectral shift and increased the fluorescence intensity. These results indicate that the amino acid insertion produces a space near the chromophore, and the side chain of the inserted residue significantly affects the environment around the chromophore.

### Effect of the number of glycine inserted into the β-barrel structure on fluorescence

**Figure 2:**
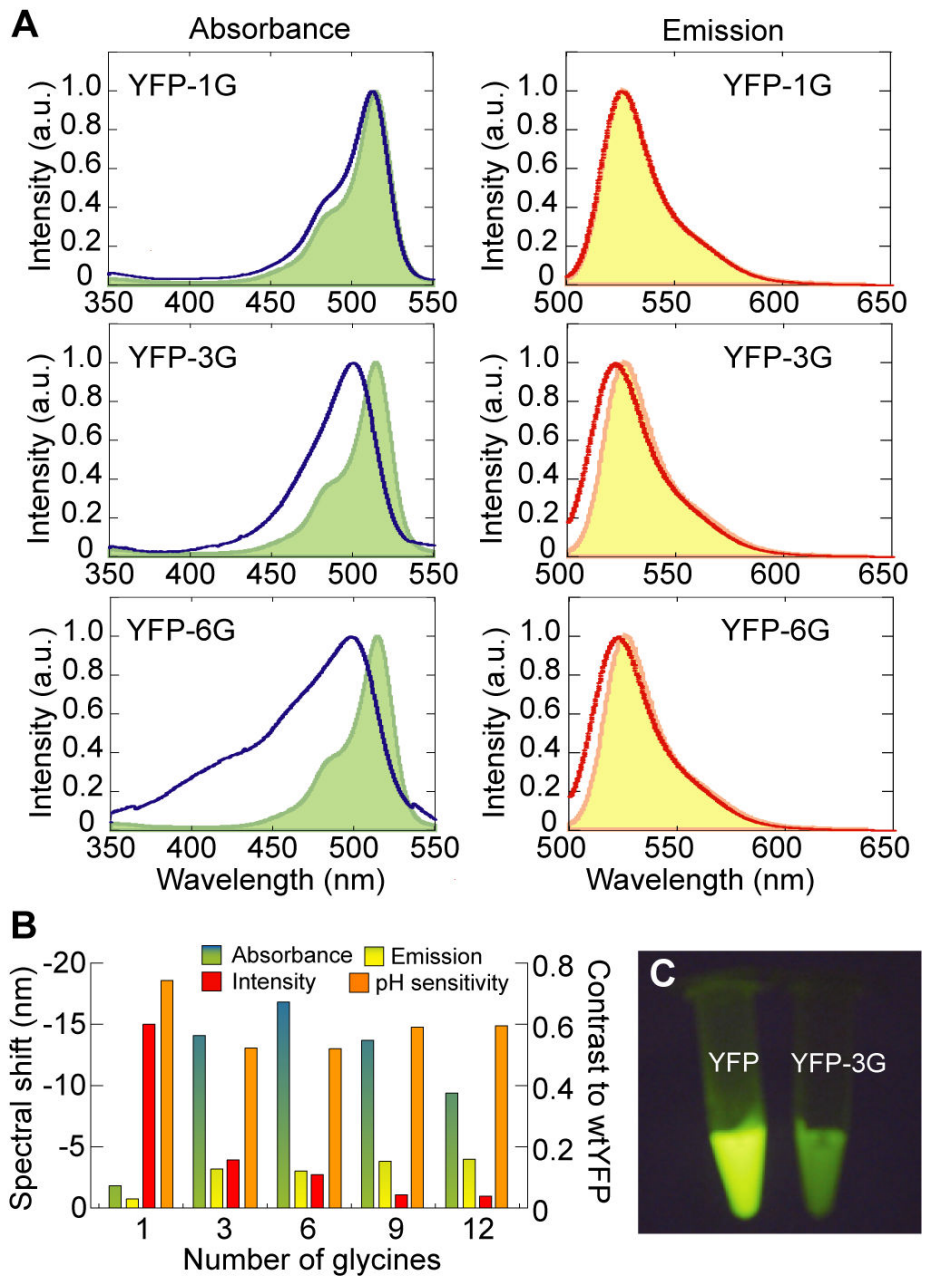
Effect of insertion of 'G' rich fragments into YFP. (A) Absorbance (blue) and emission (red) spectra of YFP-1G (upper), YFP-3G (middle), and YFP-6G (lower). Green, absorbance of YFP; yellow, emission of YFP. Emission spectra were obtained at 488 nm excitation. The intensities are normalized as to each peak intensity. (B) Spectral shifts of absorbance (green) and emission (yellow), fluorescence intensities (red), and pH dependency (orange) of YFP-nG that is defined as the ratio of the fluorescence intensities at pH 8.0 and 7.0. The intensities are normalized as to wild-type YFP. (C) Fluorescence photographs of YFP and YFP-3G solutions on blue-light (488 nm excitation) transilluminator. The concentrations of proteins were 0.33 mg/ml. We set both samples on side by side, and took the photograph simultaneously.

To further investigate the relationship between the space near the chromophore and the fluorescence characteristics, we inserted various numbers of amino acids into the same site. The inserted amino acids are mainly glycine to reduce steric hindrance and various effects by the side chain. The YFP mutants were named YFP-nG, where n is the number of the inserted amino acids (1, G; 3, GGG; 6, GGSGGT; 9, GGSGGTGGS; and 12, GGSGGTGGSGGT). The three residue insertion caused a further blue-shift in both the absorbance and emission spectra compared to the single residue insertion (Figure 2, A and B). However, an insertion of more than six residues did not cause further shift (Figure 2B, *green and yellow*). The fluorescence intensity decreased with the number of inserted residues, suggesting a greater interaction between the chromophore and water molecules and/or the structural change near the chromophore (Figure 2B, *red bars*). The fluorescence intensity of YFP-3G was 6-fold lower than that of YFP, but it was still enough to be visually detected (Figure 2C). The pH sensitivity was also increased by the glycine insertion (Figure 2B, *orange bars*, and Figure S1). The pKa estimated from the pH sensitivity was not much affected by the insertion (pKa = 6.5 for YFP, 7.0 for YFP-1G and 6.9 for YFP-3G). These results indicate that the environment around the chromophore was strongly related to the number of the inserted amino acid residues.

### Crystal structures of glycine-inserted mutants

**Figure 3:**
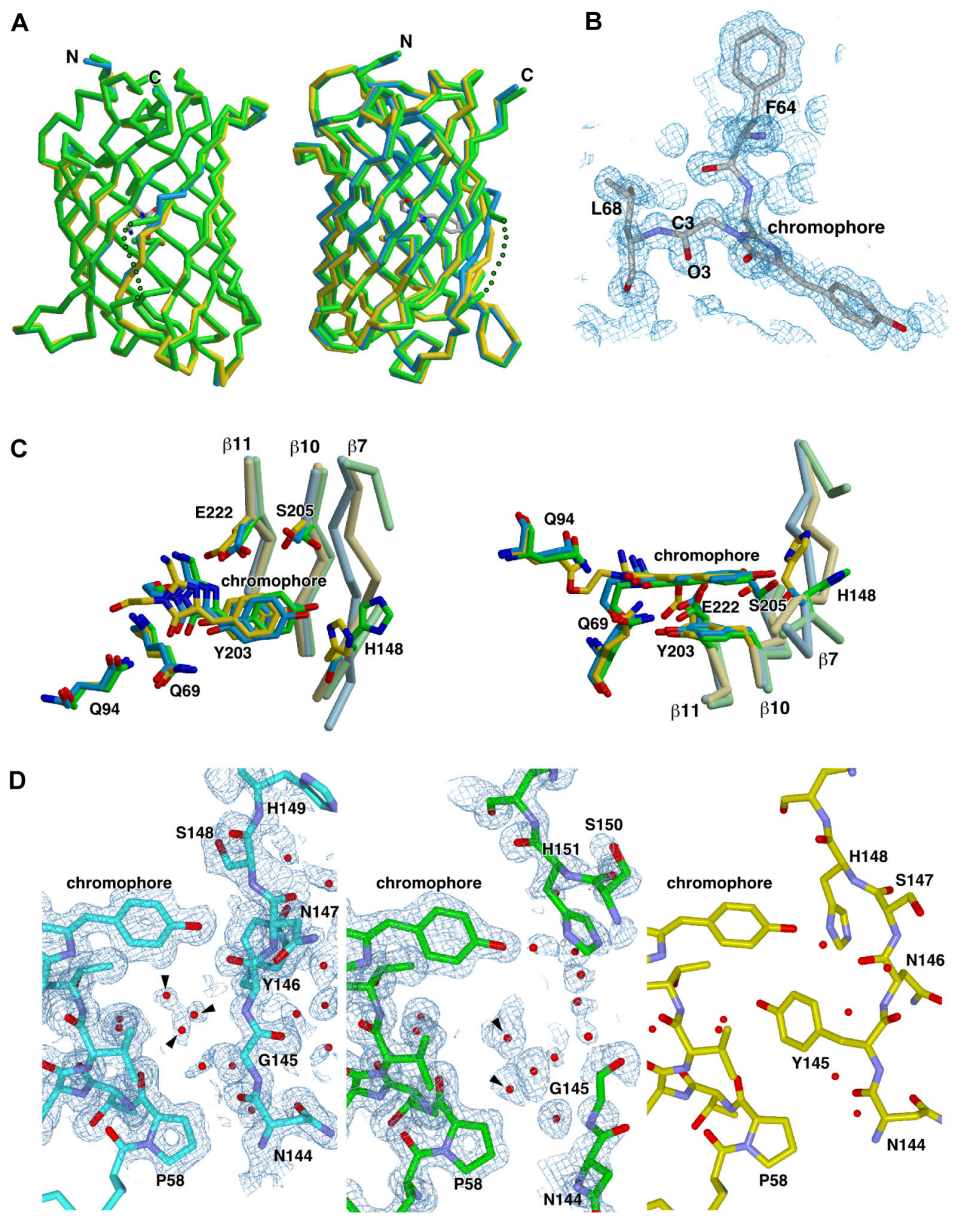
Crystal structure of YFP inserted 'G' and ‘GGG’. (A) Overlay structure diagram showing front (left) and side (right) views of YFP (yellow; PDB ID: 1yfp), YFP-1G (cyan), and YFP-3G (green). The missing residues of YFP-3G are indicated by dotted line. (B) Close-up view of the YFP-3G chromophore shown with the 2Fo-Fc density map. (C) Superposition of the structures of YFP (yellow), YFP-1G (cyan), and YFP-3G (green) around the chromophore. Residues interact with the chromophore are shown in stick model with the main-chain backbone trace of β7, β10 and β11. Oxygen and nitrogen atoms are colored in red and blue, respectively. The right panel is viewed from the bottom of the left panel. (D) Water molecules near the chromophore ring. The stick models of YFP-1G (left) and YFP-3G (middle) and YFP (right) are colored in cyan, green and yellow, respectively. YFP-1G and YFP-3G are shown with the 2Fo-Fc density map. Water molecules are represented by red ball. The arrows indicate water molecules filling the space where Tyr145 of YFP was located. Oxygen and nitrogen atoms are colored in red and blue, respectively.

To confirm that the glycine insertion produced a space near the chromophore, we determined the crystal structures of YFP-1G and YFP-3G at 1.3 Å and 1.5 Å resolution, respectively. The effect of the glycine insertion on the overall structural change is limited at around β-strand 7 (Figure 3A). In the YFP-1G structure, β-strand 7 is slightly distorted, causing a small expansion of the barrel (Figure 3A, *cyan*). In the YFP-3G structure (Figure 3A, *green*), the four amino acid residues of β–strand 7 (146-149, GGYN) were not modeled due to poor electron density in this region, suggesting that these residues exhibit conformational flexibility. The insertion of glycine induced significant conformational change of the chromophore. The O3-C3 carbonyl bond of the chromophore is flipped into the close-conformation (Figure 3B), which is also found in the structure of E2GFP, a GFP variant S65T/T203Y [23]. The O3-C3 conformation of E2GFP is flipped back to the normal open-conformation by chloride binding, and the open-conformation had the absorbance at around 400 nm while the closed-one did not [23]. The absorbance spectrum of YFP, which has the open-conformation, showed not only the main peak at 510 nm but also a small peak at 380 nm, and the small peak was not affected by the chloride ion concentration (Figure S2, A and C). On the contrary, the absorbance spectrum of YFP-3G did not show the peak at 380 nm with low chloride ion concentration, but the peak appeared when the chloride ion concentration was increased (Figure S3, B and D). This chloride ion dependency is similar to that of E2GFP [23], suggesting that YFP-3G switches its chromophore structure to the open-conformation by binding of the chloride ion. Compared with the YFP structure (PDB ID: 1yfp), the chromophore rings in YFP-1G and -3G tilted and shifted toward the surface of the barrel by approximately 0.5 Å and 0.9 Å, respectively (Figure 3C). The relative arrangement of the main chromophore and the tyrosine phenol ring was examined by aligning the structures using Tyr203 (Tyr204 for YFP-G1 and Tyr206 for YFP-G3) (Figure S3). The remarkable difference is that the plane of the main chromophore ring of the mutants tilted about 10^o^ from that of YFP. In addition, the main chromophore of YFP-3G is largely shifted. These relative movements of the chromophore probably caused the observed spectral blue-shifts. The glycine insertion shifts β-strand 7 along the chain to the N-terminal direction for YFP-3G and the C-terminal direction for YFP-1G (Figure 3D). These shifts considerably change the environment around the main chromophore of the mutants. The most remarkable difference is that the side chain of Tyr145 (Tyr146 for YFP-G1 and Tyr148 for YFP-G3) moved away from the neighborhood of the chromophore, producing a large space near the chromophore. The space is filled by water molecules in both YFP-1G and YFP-3G structures (Figure 3D, *arrow heads*). Thus the interaction between the water molecules and the chromophore probably decreased the fluorescence intensity of the mutants.

### Hydrostatic Pressure dependency of the glycine-inserted mutants

**Figure 4:**
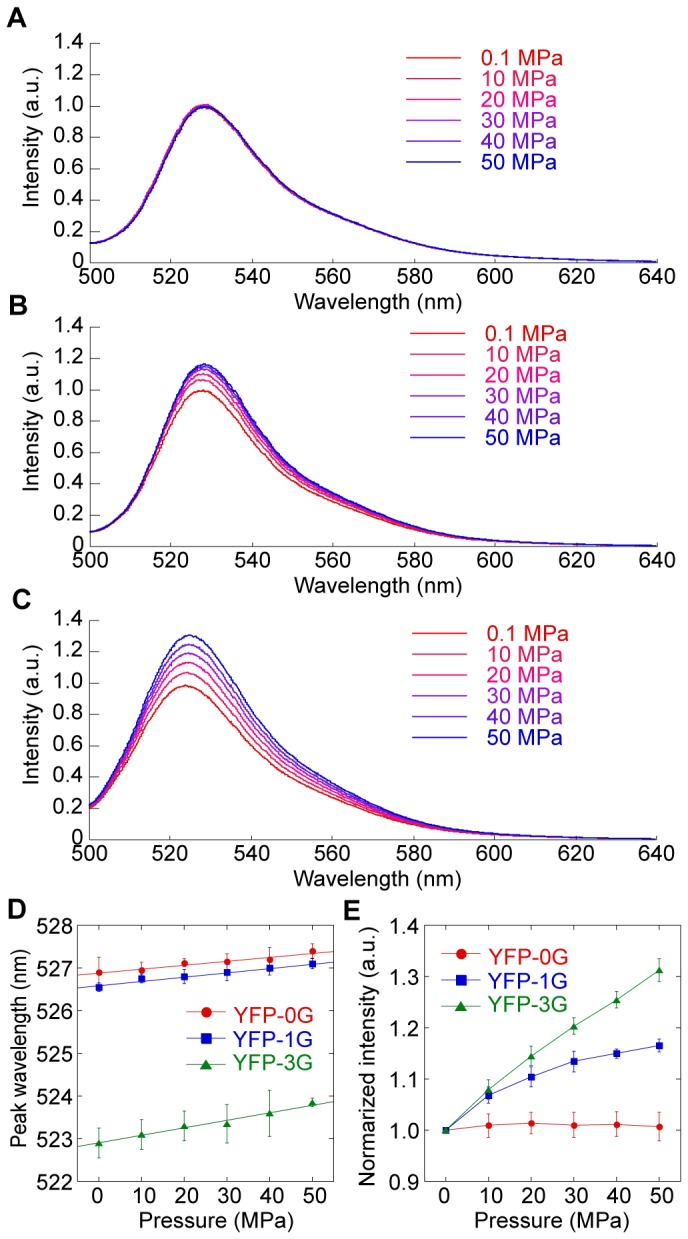
Hydrostatic pressure dependency of the YFP, YFP-1G and YFP-3G. (A, B, C) Fluorescence spectra of YFP (A), YFP-1G (B), and YFP-3G (C) between 0.1 and 50 MPa (red to blue). The traces represent the averages of six individual trials. All spectra are normalized with the spectrum at 0.1 MPa. (D) Peak shifts of the fluorescence spectra of YFP (red), YFP-1G (blue), and YFP-3G (green) between 0.1 and 50 MPa. (E) Pressure dependence of the peak fluorescence intensities of YFP (red), YFP-1G (blue), and YFP-3G (green) between 0.1 and 50 MPa. The values are normalized with the value at 0.1 MPa. All emission spectra were obtained at 488 nm excitation. Error bars, standard deviation.

As mentioned above, we were able to construct the YFP as we wanted. Next, we investigated the effects of hydrostatic pressure on the fluorescence of YFP, YFP-1G and -3G (Figure 4), and found that a pressure increase of 50 MPa caused a spectral red-shift for YFP, YFP-1G, and YFP-3G, even at room temperature (25°C) (Figure 4 A–D). The spectral shift can be easily detectable at higher pressure (<300 MPa) (Figure S4). At pressure levels of less than 300 MPa, the extent of the spectral shift for YFP-3G resembled those for YFP and YFP-1G (Figure S4D). This indicates that the entry of water molecules with the glycine insertions due to pressure is not responsible for the rearrangement of Tyr203 and the chromophore [21,22]. Nevertheless, there is an obvious difference in the responses to hydrostatic pressure between YFP, YFP-1G and YFP-3G (Figure 4E). While YFP did not show the pressure dependency in fluorescent intensity below 50 MPa (Figure 4E, *red*), the single glycine insertion made YFP sensitive to the pressure (blue), and the further glycine insertion enhanced its pressure dependency (green). At the higher pressure, the intensity of YFP decreased with increased pressure, while that of YFP-3G increased and that of YFP-1G was biphasic (Figure S4E). The pressure sensitivity of YFP-3G was improved 5-fold at 300 MPa and over 13-fold at 100 MPa, compared with that of YFP. These results indicate that the hydrostatic pressure dependency of the glycine inserted mutants showed the different manner among YFP, YFP-1G and YFP-3G, and YFP-3G showed the best performance as pressure sensor. Though the YFP could not be applied for pressure sensor below 50 MPa, the YFP-3G works well.

We then investigated whether YFP-3G sense osmotic pressure or not by measuring the dependency of YFP, YFP1G and YFP3G on sucrose, which is a crowding agent often used to change osmotic pressure [24]. Increasing the sucrose concentration decreased the fluorescent intensity of YFP (Figure S5 A and C, *red*), indicating the slight dependence of YFP on osmotic pressure. The three glycine insertion diminished the sucrose dependency (Figure S5 C and C, *blue*), indicating that YFP-3G does not sense osmotic pressure. Thus, the glycine insertions enhanced the sensitivity to hydrostatic pressure apart from osmotic pressure.

### Intracellular pressure measurement

**Figure 5:**
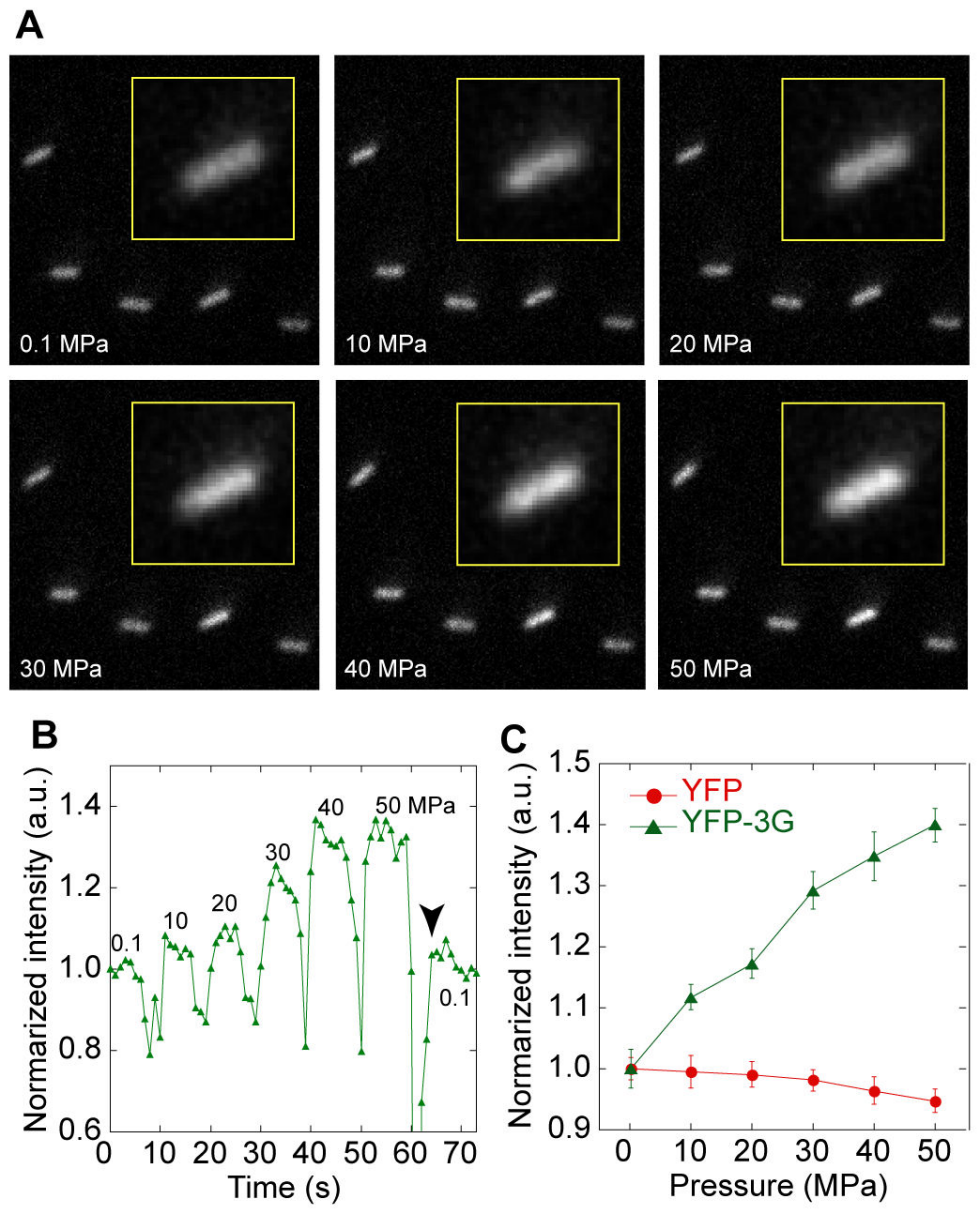
Fluorescence intensity change of YFP-3G in *E. coli* with increase of hydrostatic pressure. (A) Fluorescence image of *E*. *coli* cells expressing YFP-3G at 0.1, 30 and 50 MPa. Insertions are the enlarged images of the single *E*. *coli* cell. (B) Time curse of fluorescent intensity of single *E*. *coli* expressing YFP-3G with the change of hydrostatic pressure. Values are the applied pressure. (C) Pressure dependence of the fluorescence intensities of *E*. *coli* expressing YFP (red) and YFP-3G (green) at 0.1-50 MPa (N = 74-151). Error bars, standard error.

To further confirm that the fluorescence intensity of YFP-3G changes with hydrostatic pressure in living cells, we conducted a model experiment with *E. coli*. We previously constructed a fluorescent microscope with a high-pressure chamber, which enables to obtain fluorescent images while increasing the hydrostatic pressure [25]. When pressure was increased at 10 MPa interval using a hand-pump, we could pursue the fluorescent change of a single *E. coli* cell though the intensity temporally decreased just before the pressure increase due to defocusing (Figure 5 A and B). The fluorescent intensity in the cell was reversed by releasing the pressure (Figure 5B, *arrowhead*). Fluorescent intensities of YFP-3G expressed in *E. coli* cells increased with the hydrostatic pressure while those of YFP slightly decreased (Figure 5C). Though the YFP-3G detected not only pressure but also temperature (Figure S6), because the temperature was kept constant in this assay, this result clearly indicates that the fluorescence intensity change of YFP-3G is usable for pressure detection in a living cell.

## Discussion

We here have shown the design, construction and model experiment of the pressure sensitive fluorescent protein. Based on the previous design of the fluorescent protein-based sensors, such as the circular permutation [7,9,26–28], we focused on the Tyr145 (Figures 1 and 2) to make a space for water molecules near the chromophore (Figure 3). Insertion of a few glycine residues between Asn144 and Tyr145 enhanced the pressure dependency of the fluorescent intensity of YFP (Figures 4 and 5).

The invasion of the water molecules into the β-can structure was confirmed by crystal structure analysis (Figure 3). We fitted the electron density between G145 and H151 in YFP-3G with water molecules (Figure 3D, meddle), as using the protein backbone instead would have resulted in accommodating only two of the four residues in the β–strand 7 because of disorder. Although not shown, there are weak disordered densities on the right of the middle panel of Figure 3D. These densities probably correspond to a disordered chain. Moreover, an obvious difference in the electron density of the water molecules in the cavities of YFP-1G and YFP-3G was observed (Figure 3D). Because the four residues of YFP-3G were disordered, the cavity of YFP-3G would be partially exposed to the outside, which may change the arrangement of the water molecules. These water molecules were therefore considered not fixed in the cavity, and their densities were more dispersed in YFP-3G than in YFP-1G. Thus, it was speculated that the water molecules in the cavity in YFP-3G have higher mobility than those in YFP-1G.

The hydrostatic pressure dependency of fluorescence for small molecules has been explained by the interaction of water molecules and the chromophore. In general, pressure increase enhances the water-chromophore interaction that quenches the fluorescence [29]. However, the fluorescence intensity of YFP-3G was increased with pressure, and that of YFP-1G was biphasic (Figure S4E). Thus the hydrostatic pressure dependency of YFPs cannot be explained with the simple water-chromophore interaction model alone. The structural analysis of YFP-1G and YFP-3G showed that water molecules fill the space where Tyr145 was located in YFP and interact with the chromophore (Figure 3D). These water molecules may be discharged from the β-barrel structure by increasing the hydrostatic pressure, resulting in the increase in fluorescent intensity. While YFP and YFP-3G showed the opposite responses in fluorescence intensity to hydrostatic pressure (Figures 4 and S4), they showed the same response to the temperature (Figure S6). Moreover, the dependency of YFP-1G in the hydrostatic pressure was biphasic while that in the temperature was monophasic. YFP actually showed a biphasic pressure dependency at low pressure range (<50 MPa), although the intensity change was small (Figure S4). This suggests that the biphasic pressure dependency is an intrinsic property of YFP, and that the flexion point shifts toward higher pressure with the space near the chromophore made by glycine insertion.

The importance of pressure in biological processes has been indicated by several reports. It is known that the hydrostatic pressure affects biological behaviors, for example, the acceleration of egg activation requires up to 50 MPa [30]. This value is large enough to be detected with YFP-3G. However, many biological events are occurring in lower hydrostatic pressure, for instance, the plant hoists water using the difference of the osmotic pressure between the adjacent cells which was estimated to be less than 1 MPa [31]. The intensity change of YFP-3G was 1% with the pressure change of 1 MPa. The further improvement of sensitivity of YFP-3G is awaited.

To summarize, though further investigation of the mechanism underlying the amino acid insertions remain to be investigated, we found that glycine insertions causes the new static water interaction to the chromophore, which can enhance the sensitivity of fluorescent protein to the hydrostatic pressure. Along with these observations, the present engineering method for fluorescent proteins, amino acid insertion, has been first reported as to challenge to develop a protein indicator to sense intracellular pressure. We believe that further study will unveil a fundamental biomechanisms that no one has previously seen, that is, the relationship between water and biomolecules in physiological conditions.

## Materials and Methods

### Construction and purification of YFP-inserted glycine-rich fragments

We used YFP that contain four amino acids substitutions in GFP as previously reported [5], but not Citren (Q69M mutant of YFP) [32]. The YFP cDNA clone was inserted into *E. coli* expression vector pET-T7 between its NdeI and HindIII sites. The cDNAs of YFP-1G, -3G, and -6G were obtained as PCR products by using primers that included G, GGG, and GGSGGT, respectively. We added serine and threonine into the glycine-rich chain to avoid forming a secondary structure, because they prevent internalization into the hydrophobic core by making the chain more soluble [33]. The GGSGGT sequence has been widely used as flexible linker in FRET indicators (7). After reinserting YFP-6G cDNA into the pET-T7 vector, YFP-9G and YFP-12G was obtained by inserting annealed oligo nucleotides (5’-aggaagtac-3’ and 5’-ttcctgtac-3’ for YFP-9G, 5’-aggaagtacaggaagtac-3’ and 5’-ttcctgtacttcctgtac-3’ for YFP-12G) at the KpnI site of YFP-6G cDNA. For protein purification, YFP, -1G, -3G, -6G, -9G and -12G were ligated into a pAL7 vector (BIO-RAD) between its HindIII and NcoI sites, and then transformed into *E. coli*, Rossetta2 (DE3).

We used a Profinity eXactTM fusion-tag system (BIO-RAD) to purify tag-free proteins containing its native N-terminal amino acid sequence. The YFP, -1G, -3G, -6G, -9G and 12G proteins were overproduced in *E. coli*, and purified by using Profinity eXact purification resin with Bio-Scale Mini Profinity eXact cartridges following the protocol recommended by BIO-RAD. The obtained amounts of protein with Tonein-TP (Otsuka Pharmaceutical. Co. Ltd, Japan) were 7.5 mg for YFP, 5.0 mg for YFP-1G, 4.8 mg for YFP-3G, 0.06 mg for YFP-6G, 0.05 mg for YFP-9G and 0.03 mg for YFP-12G.

### Structure determination of YFP-inserted glycine-rich fragments

Initial crystallization screening using screening kits (Wizard I and II, Cryo I and II (Emerald Bio structures), and Crystal Screen I and II) was carried out by the sitting-drop vapor-diffusion technique. Thin needle crystals of YFP-1G and YFP-3G grew within a week under various conditions at 277 K. The conditions were then optimized by varying the precipitant concentration, pH and additive concentration using the hanging-drop method with the micro seeding technique at 277 K. Crystals suitable for X-ray measurement were obtained from drops prepared by mixing 1.5 ml of a protein solution (10-13 mg ml^-1^) with 1.5 ml of a reservoir solution comprising 100 mM Bis-tris-HCl pH 6.5, 13.5% PEG8000 and 350 mM calcium acetate for YFP-1G, and 100 mM Bis-tris-HCl, pH 6.5, 17% PEG8000, and 200 mM calcium acetate for YFP-3G. YFP-1G crystals were grown in the space group of P212121, with unit cell dimensions of a = 51.2, b = 62.8, c = 69.8 Å, and diffracted up to 1.3 Å. YFP-3G crystals also belong to space group P 212121, with unit cell dimensions of a = 51.1, b = 63.0, c = 70.0 Å, and diffracted to 1.5 Å.

X-ray diffraction data were collected at the wavelength of 1.0 Å at SPring-8 beamline BL38B1 (Harima, Japan). Crystals were soaked in a mixture comprising 90% (v/v) of the reservoir solution and 10% (v/v) of MPD for a few seconds, and then immediately transferred to liquid nitrogen. The diffraction data were recorded on an ADSC Quantum 210 CCD detector (Area Detector Systems Corporation) under a cold nitrogen gas flow at 100 K. The diffraction data were processed and scaled using programs MOSFLM [34] and SCALA in the CCP4 program suite [35], respectively.

The structures of YFP-1G and YFP-3G were determined by the molecular replacement method using MOLREP (Collaborative Computational Project Number 4, 1994) with the structure of YFP citrine (PDB ID 3DQ7) as a search model. The model was refined using program CNS [36]. After single round of refinement, the model was modified with Coot [37] and refined again. During the refinement process, iterative manual modifications were performed using an ‘omit map’. The structure of YFP-1G was refined to an R factor of 19.6% and a free R factor of 21.1% at 1.3 Å resolution, and the structure of YFP-3G to an R factor of 19.1% and a free R factor of 20.8% at 1.5 Å resolution. The Ramachandran plots of YFP-1G and YFP-3G showed 90.8% and 91.7% residues to be located in the core region, respectively, with 9.2% and 8.3% residues in the allowed region, respectively. Data collection and refinement statistics are summarized in Table 1. The coordinates of YFP-1G and YFP-3G have been deposited in PDB under accession numbers 3VGQ (YFP-1G) and 3VGR (YFP-3G), respectively.

**Table 1:** Data collection and refinement statistics (molecular replacement).

		YFP-1G	YFP-3G
**Data collection**			
Space group		*P*2_1_2_1_2_1_	*P*2_1_2_1_2_1_
Cell dimensions			
	*a*, *b*, *c* (Å)	51.2, 62.8, 69.8	51.1, 63.0, 70.0
Resolution (Å)		17.25 - 1.30	21.28 - 1.50
		(1.37 - 1.3) *	(1.59 - 1.5)
*R*_merge_		6.5 (46.9)	6.6 (41.5)
*I* / σ*I*		15.9 (3.5)	17.5 (3.4)
Completeness (%)		97.3 (94.5)	98.6 (91.6)
Redundancy		5.6 (5.3)	6.5 (4.4)
**Refinement**			
Resolution (Å)		17.25 - 1.30	21.28 - 1.50
		(1.38 - 1.3)	(1.59 - 1.5)
No. reflections		54393 (8252)	36294 (5276)
*R*_work_ / *R*_free_		19.8/21.2 (27.3/26.9)	19.2/20.8 (24.9/27.7)
No. atoms			
	Protein	1833	1812
	Ligand/ion	0	0
	Water	410	355
*B*-factors			
	Protein	11.8	13.4
	Water	27.6	28.3
R.m.s. deviations			
	Bond lengths (Å)	0.008	0.006
	Bond angles (°)	1.7	1.5

All values in parentheses are for the highest-resolution shell.

* Number of xtals for each structure should be noted in footnote.

### Absorbance and fluorescence spectra measurements

The YFP, -1G, -3G, -6G and -12G proteins were diluted to 0.1-0.3 mg/ml in 20 mM Hepes-NaOH (pH 8.0), and then scanned for absorbance between 250 and 600 nm (Shimadzu UV–Vis Spectrophotometer UV-1650PC). Fluorescence measurements were carried out on a Hitachi RF5300-PC fluorescence spectrophotometer with a protein concentration of 0.1-0.3 mg/ml in 20 mM Hepes-NaOH (pH 8.0). The excitation wavelength was set to 488 nm. The emission was scanned between 500 and 650 nm, and the emission peak wavelength was estimated by the zero crossing method.

### Fluorescence measurements at high pressure

A high-pressure optical chamber (PCI500, Syn Corporation, Japan) was set inside a Hitachi RF5300-PC fluorescence spectrophotometer. By using a high-pressure pump (HP-500, Syn Corporation, Japan), hydrostatic pressure was increased slowly (approximately 5 MPa/s) to avoid any temperature increase. One minute after reaching the desired pressure, the emission was scanned between 500 and 650 nm. The emission peak wavelength was estimated by the zero crossing method as above. The excitation wavelength was set to 488 nm. The protein concentrations were 3.3 μg/ml for YFP, 10 μg/ml for YFP-1G, and 10 μg/ml for YFP-3G in 20 mM Hepes-NaOH (pH 8.0). Experiments were performed at room temperature (25°C). Photobleaching was corrected for by applying an exponential bleaching curve obtained before the assays even the effect of the photobleaching is quite small.

### Fluorescence measurement of E. coli with high-pressure chambers

The microscopy system consisted of an epi-fluorescent microscope (Ti-E, Nikon, Japan), an objective (NA 0.6, working distance ~3 mm; CFI ELWD ADM40xC, Nikon, Japan), and an electron multiplier type charge-coupled device camera (Ixon DV887, Andor Technology, UK). An ~30 × 30 μm^2^ area was illuminated by a blue (488 nm) laser (EXLSR-488, Spectra-Physics, USA). A high-pressure chamber (HPC; Sasahara Giken, Kyoto, Japan) was mounted on the stage of the microscope [25]. The fluorescent images were obtained at 0.1-50 MPa.

For microscopic observations, *E. coli* cells expressing YFP or YFP-3G were fixed on glass surface of the chamber by a poly-lysine coat. The solution of 100 mM Hepes (pH 7.2) and 0.5% NaCl were loaded into the chamber 5 min after the fixation of the cells. The chamber was sealed and connected to a hand-pump. The hydrostatic pressure was increased by the hand-pump, which took few seconds to increase the pressure by 10 MPa.

## Supporting Information

Figure S1 **pH dependencies of YFP, YFP-1G and YFP-3G fluorescence.** (**A**, **B**, **C**) pH dependencies of YFP (**A**), YFP-1G (**B**), and YFP-3G (**C**). The intensity is normalized as to that of YFP at pH 8.0. (**D**) Summary of pH dependencies of the peak fluorescence intensities of YFP (red), YFP-1G (blue) and YFP-3G (green). The intensity is normalized as to that of each value at pH 8.0. Solid lines are the fitting curve with following equation: *F* = *A* + *B* / [1 + 10^nH(pKa-pH)^], where pKa is pH at 50% maximum, nH is Hill coefficient, and parameters A and B are related to signal baseline (*). The estimated pKa and nH are 6.5 and 1.3 for YFP, 7.0 and 1.0 for YFP-1G, and 6.9 and 1.5 for YFP-3G, respectively. All emission spectra were obtained at 488 nm excitation.
(*) Kneen M, Farinas J, Li Y, & Verkman AS. Green fluorescent protein as a noninvasive intracellular pH indicator. Biophys J. 74:1591-1599 (1998).
(TIF)

Figure S2 **Chloride dependencies of YFP and YFP-3G absorbance.** (**A**, **B**) KCl concentration dependencies of absorbance spectra of YFP (**A**) and YFP-3G (**B**). The intensity is normalized with the value at 280 nm. (**C**, **D**) Spectra difference of YFP (**C**) and YFP-3G (**D**) corresponding to spectrum at 0 mM KCl.
(TIF)

Figure S3 **Comparison of the chromophore structures of YFP, YFP-1G and YFP-3G.** YFP-1G (cyan) and YFP-3G (green) were superimposed on YFP (yellow; PDB ID: 1yfp) by fitting the side chain atoms of Tyr203 (Tyr204 for YFP-G1 and Tyr206 for YFP-G3). The right panel is viewed from the right of the left panel.
(PNG)

Figure S4 **High hydrostatic pressure dependency of the YFP, YFP-1G and YFP-3G.** (**A**, **B**, **C**) Fluorescence spectra of YFP (**A**), YFP-1G (**B**), and YFP-3G (**C**) at 0.1 (red), 100 (light magenta), 200 (magenta), and 300 (blue) MPa. The traces represent the averages of six individual trials. All spectra are normalized with the spectrum of YFP at 0.1 MPa. (**D**) Peak shifts of the fluorescence spectra of YFP (red), YFP-1G (blue), and YFP-3G (green) from 0.1 to 300 MPa. (**E**) Pressure dependence of the peak fluorescence intensities of YFP (red), YFP-1G (blue), and YFP-3G (green) from 0.1 to 300 MPa. The values are normalized with the value at 0.1 MPa. All emission spectra were obtained at 488 nm excitation. Error bars, standard deviation.
(TIF)

Figure S5 **Sucrose dependencies of YFP, YFP-1G and YFP-3G fluorescence.** (**A**, **B**, **C**) Sucrose dependencies of YFP (**A**), YFP-1G (**B**), and YFP-3G. (**C**) The intensity is normalized as to that of YFP at 0%. (**D**) Summary of sucrose dependencies of the peak fluorescence intensities of YFP (red), YFP-1G (blue) and YFP-3G (green). The intensity is normalized as to that of each sample at 0%. All emission spectra were obtained at 488 nm excitation.
(TIF)

Figure S6 **Temperature dependencies of YFP, YFP-1G and YFP-3G fluorescence.** (**A**, **B**, **C**) Temperature dependencies of YFP (**A**), YFP-1G (**B**), and YFP-3G. (**C**) The intensity is normalized as to that of YFP at 25^o^C. (**D**) Summary of temperature dependencies of the peak fluorescence intensities of YFP (red), YFP-1G (blue) and YFP-3G (green). The intensity is normalized as to that of each sample at 25^o^C. All emission spectra were obtained at 488 nm excitation.
(TIF)
