# Supplementary figures and images for: Glycine Insertion Makes Yellow Fluorescent Protein Sensitive to Hydrostatic Pressure
Source: PLoS One. 2013 Aug 27;8(8):e73212. doi: 10.1371/journal.pone.0073212 (PMC3754940; doi:10.1371/journal.pone.0073212)

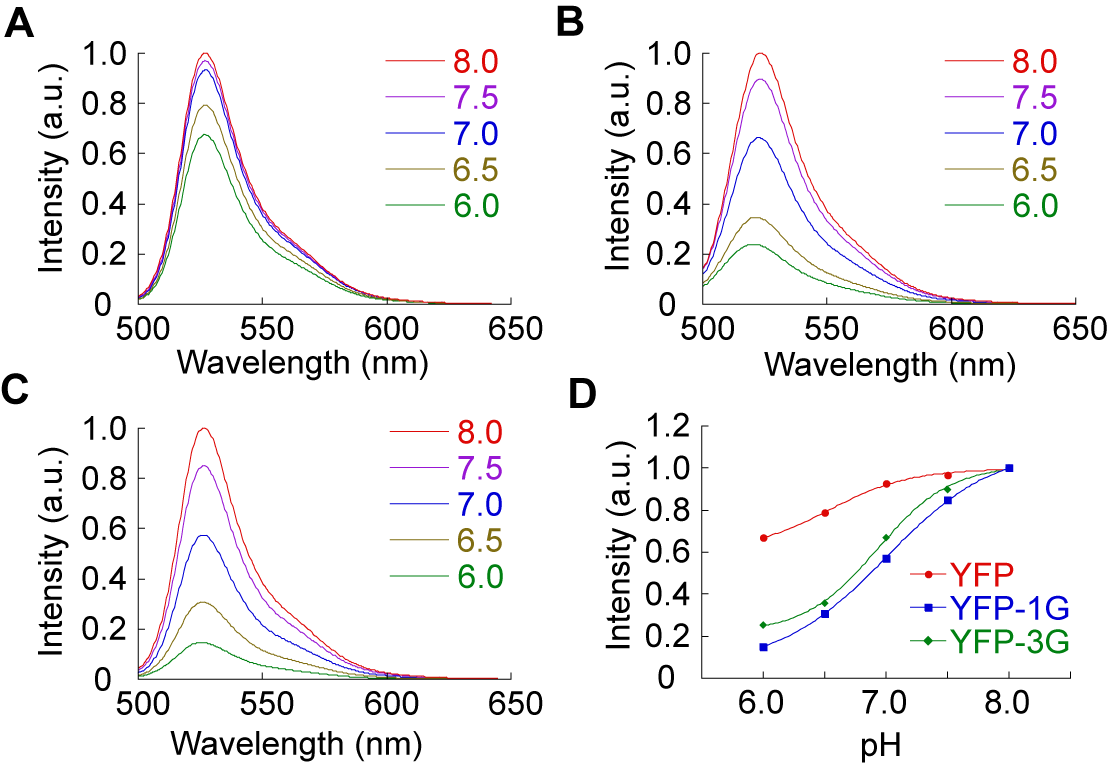

Supplement: Figure S1 — pH dependencies of YFP, YFP-1G and YFP-3G fluorescence. (A, B, C) pH dependencies of YFP (A), YFP-1G (B), and YFP-3G (C). The intensity is normalized as to that of YFP at pH 8.0. (D) Summary of pH dependencies of the peak fluorescence intensities of YFP (red), YFP-1G (blue) and YFP-3G (green). The intensity is normalized as to that of each value at pH 8.0. Solid lines are the fitting curve with following equation: F = A + B / [1 + 10nH(pKa-pH)], where pKa is pH at 50% maximum, nH is Hill coefficient, and parameters A and B are related to signal baseline (*). The estimated pKa and nH are 6.5 and 1.3 for YFP, 7.0 and 1.0 for YFP-1G, and 6.9 and 1.5 for YFP-3G, respectively. All emission spectra were obtained at 488 nm excitation. (*) Kneen M, Farinas J, Li Y, & Verkman AS. Green fluorescent protein as a noninvasive intracellular pH indicator. Biophys J. 74:1591-1599 (1998). (TIF) [file pone.0073212.s001.tif]

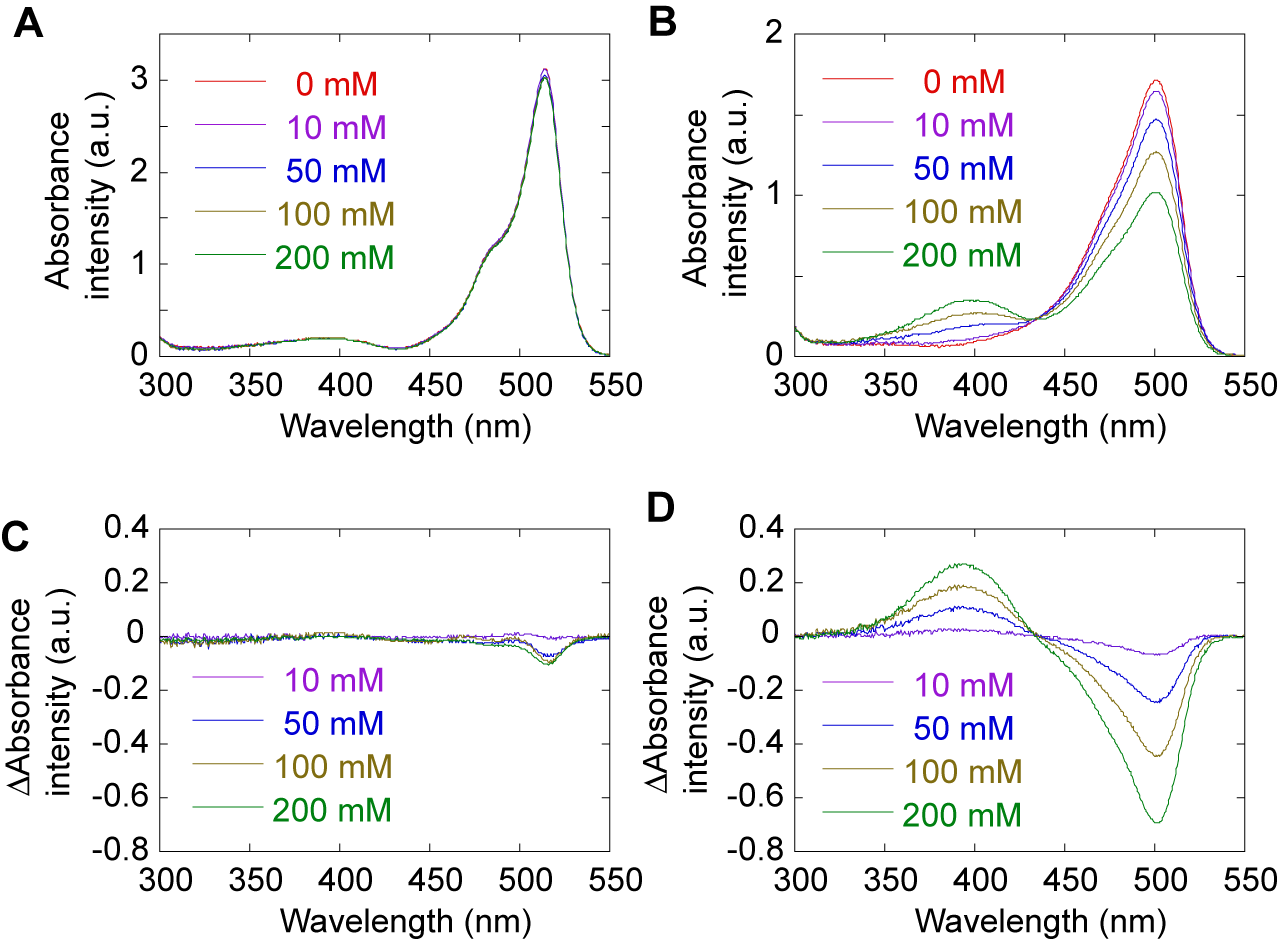

Supplement: Figure S2 — Chloride dependencies of YFP and YFP-3G absorbance. (A, B) KCl concentration dependencies of absorbance spectra of YFP (A) and YFP-3G (B). The intensity is normalized with the value at 280 nm. (C, D) Spectra difference of YFP (C) and YFP-3G (D) corresponding to spectrum at 0 mM KCl. (TIF) [file pone.0073212.s002.tif]

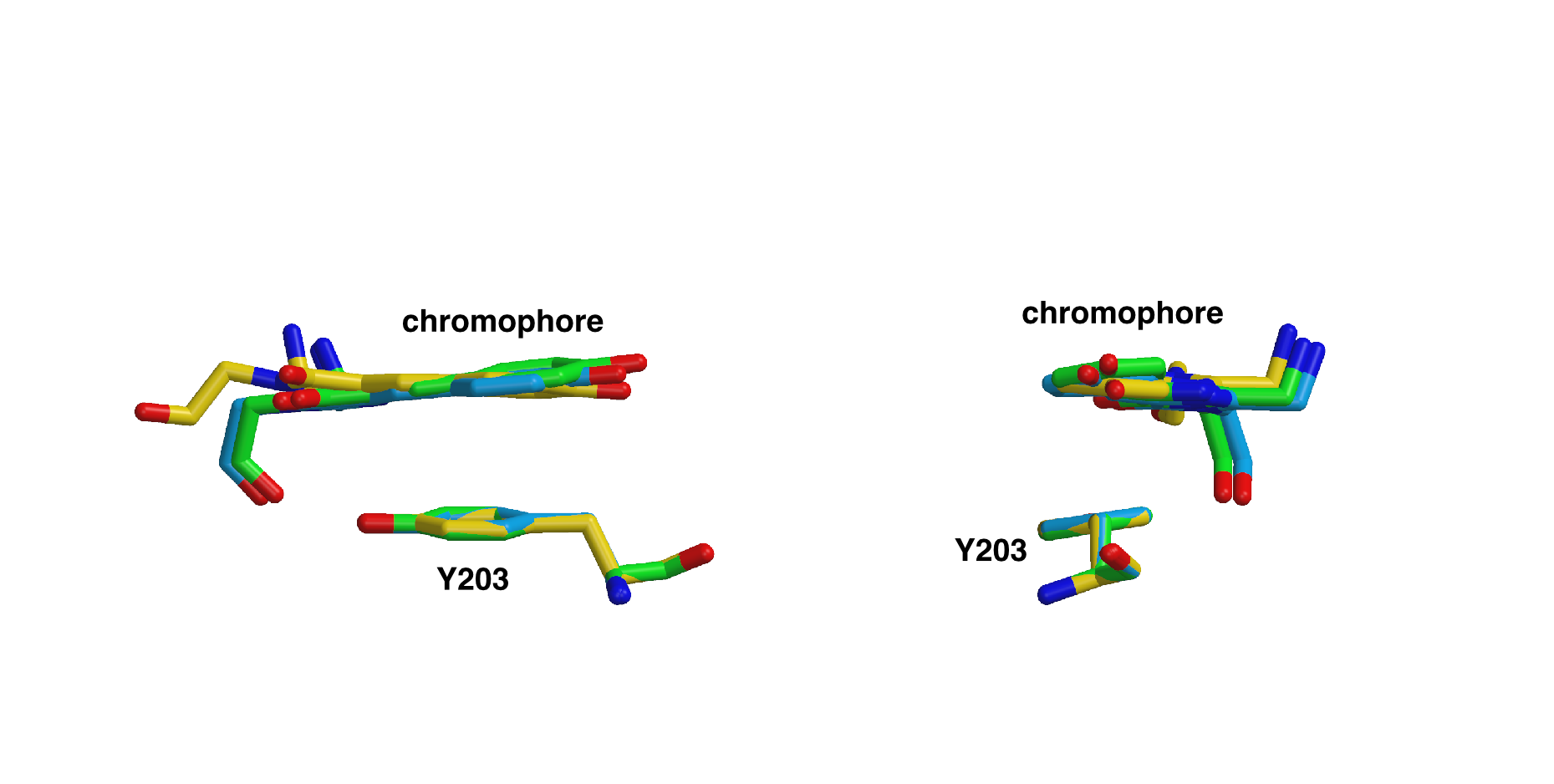

Supplement: Figure S3 — Comparison of the chromophore structures of YFP, YFP-1G and YFP-3G. YFP-1G (cyan) and YFP-3G (green) were superimposed on YFP (yellow; PDB ID: 1yfp) by fitting the side chain atoms of Tyr203 (Tyr204 for YFP-G1 and Tyr206 for YFP-G3). The right panel is viewed from the right of the left panel. (PNG) [file pone.0073212.s003.png]

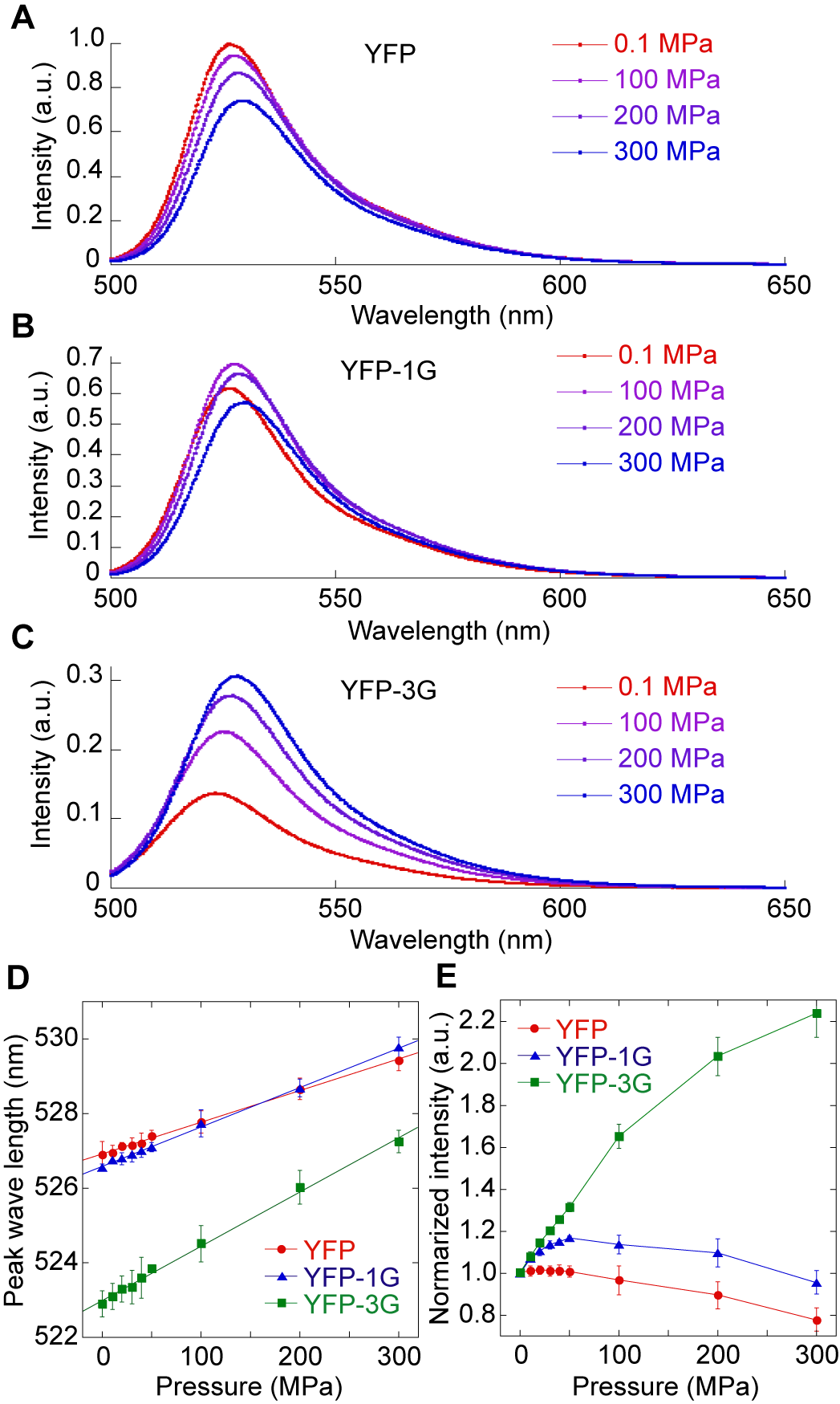

Supplement: Figure S4 — High hydrostatic pressure dependency of the YFP, YFP-1G and YFP-3G. (A, B, C) Fluorescence spectra of YFP (A), YFP-1G (B), and YFP-3G (C) at 0.1 (red), 100 (light magenta), 200 (magenta), and 300 (blue) MPa. The traces represent the averages of six individual trials. All spectra are normalized with the spectrum of YFP at 0.1 MPa. (D) Peak shifts of the fluorescence spectra of YFP (red), YFP-1G (blue), and YFP-3G (green) from 0.1 to 300 MPa. (E) Pressure dependence of the peak fluorescence intensities of YFP (red), YFP-1G (blue), and YFP-3G (green) from 0.1 to 300 MPa. The values are normalized with the value at 0.1 MPa. All emission spectra were obtained at 488 nm excitation. Error bars, standard deviation. (TIF) [file pone.0073212.s004.tif]

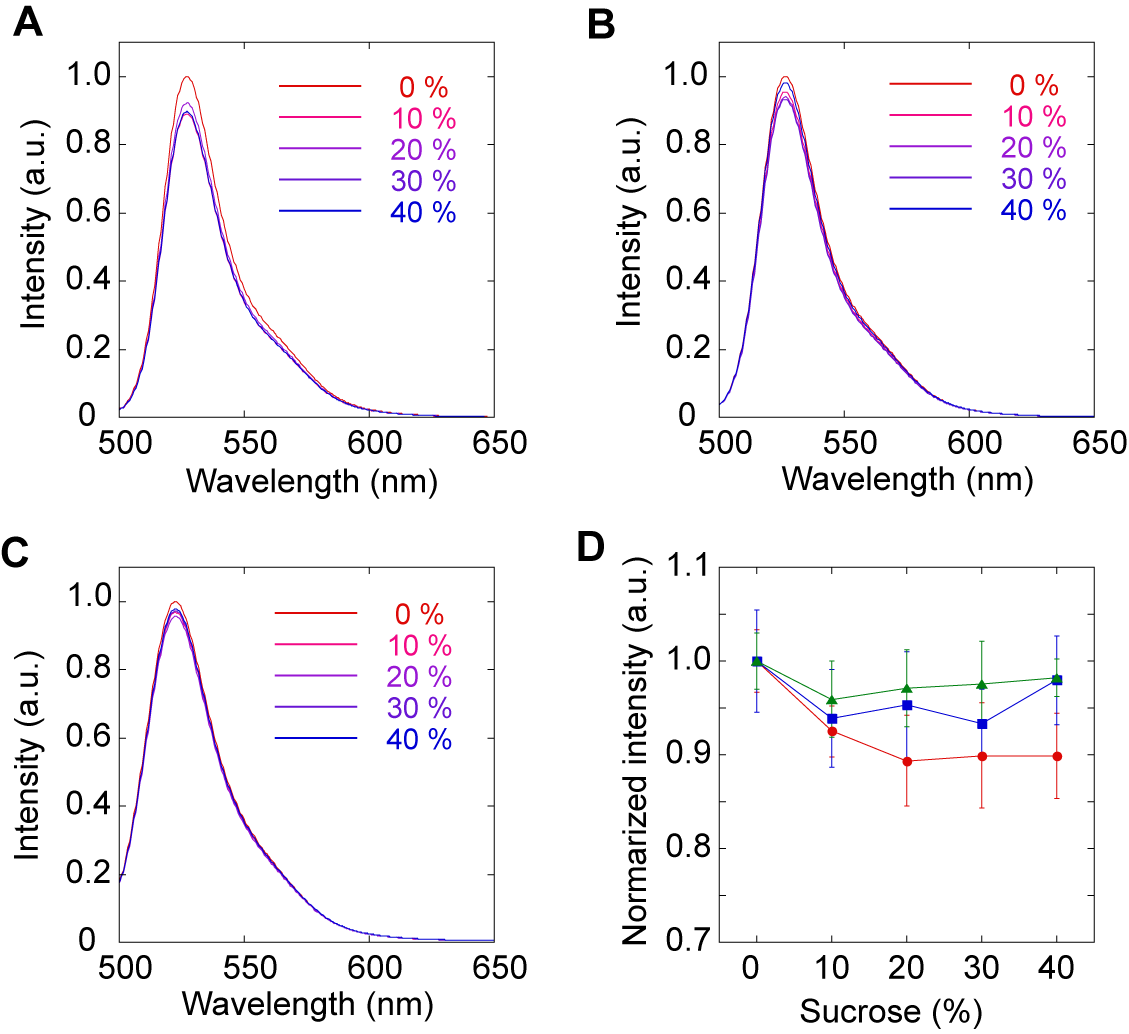

Supplement: Figure S5 — Sucrose dependencies of YFP, YFP-1G and YFP-3G fluorescence. (A, B, C) Sucrose dependencies of YFP (A), YFP-1G (B), and YFP-3G. (C) The intensity is normalized as to that of YFP at 0%. (D) Summary of sucrose dependencies of the peak fluorescence intensities of YFP (red), YFP-1G (blue) and YFP-3G (green). The intensity is normalized as to that of each sample at 0%. All emission spectra were obtained at 488 nm excitation. (TIF) [file pone.0073212.s005.tif]

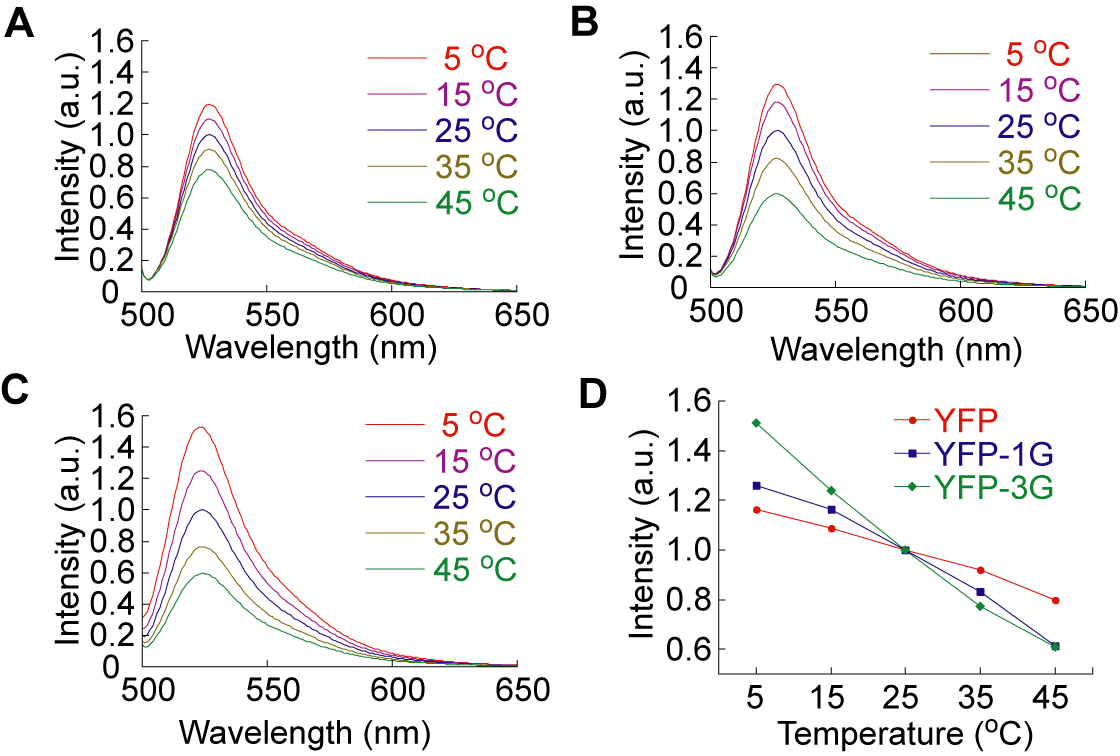

Supplement: Figure S6 — Temperature dependencies of YFP, YFP-1G and YFP-3G fluorescence. (A, B, C) Temperature dependencies of YFP (A), YFP-1G (B), and YFP-3G. (C) The intensity is normalized as to that of YFP at 25oC. (D) Summary of temperature dependencies of the peak fluorescence intensities of YFP (red), YFP-1G (blue) and YFP-3G (green). The intensity is normalized as to that of each sample at 25oC. All emission spectra were obtained at 488 nm excitation. (TIF) [file pone.0073212.s006.tif]
